# Acute Spinal Cord Injury in the Setting of C6 Pathologic Fracture Secondary to Metastatic Paraganglioma: A Case Report and Review of Literature

**DOI:** 10.7759/cureus.98403

**Published:** 2025-12-03

**Authors:** Arshi Kaur, Sarah Danehower, Hannah G Rupp, Ramin Hamidi, Mercia J Bezerra Gondim, Thomas Altstadt

**Affiliations:** 1 Neurological Surgery, University of Louisville, Louisville, USA; 2 Diagnostic Radiology, University of Louisville, Louisville, USA; 3 Pathology and Laboratory Medicine, University of Louisville, Louisville, USA

**Keywords:** cervival metastasis, malignant, paraganglioma, pathologic fracture, rare

## Abstract

Paragangliomas (PG) are rare extra-adrenal neuroendocrine tumors that arise from neural crest-derived chromaffin cells. Metastatic involvement of the spine, particularly in the cervical region, is exceptionally uncommon. Here, we present an exceedingly rare case of a 25-year-old male with metastatic spread from a recurrent functional aortocaval paraganglioma to the cervical spine, ultimately leading to a pathological fracture, acute spinal cord injury, and neurogenic shock requiring urgent intervention.

## Introduction

Paragangliomas (PGs) are rare neuroendocrine neoplasms arising from chromaffin cells of neural crest progenitors located extra-adrenally [1]. PGs account for 0.3% of all neoplasms and typically occur in the fifth or sixth decade of life [2-4]. 10% of PGs are malignant [5] and are diagnosed by either local recurrence after resection of the primary mass, or findings of distant metastasis [3,6]. Metastatic lesions include bone, lymph nodes, lung, and liver [1,7]. Metastasis to the vertebrae is extremely rare [3,8-9], with cervical metastasis being the rarest of all [3,6]. Isolated reports identify the presence of cervical PG [3,6], but only one other case, to the best of our knowledge, has resulted in pathologic fracture with acute quadriparesis [10].

Here we present an exceedingly rare case of a 25-year-old male with metastasis of a recurrent functional aortocaval PG to the C5-C7 vertebrae, ultimately resulting in a fracture and subsequent acute spinal cord injury with neurogenic shock.

## Case presentation

A 25-year-old male with no reported past medical history presented to the emergency department with neck pain and ASIA B spinal cord injury at the C6 level after falling five feet off the back of a truck. Patient denied loss of consciousness, or extreme flexion or extension of his neck.

**Figure 1:**
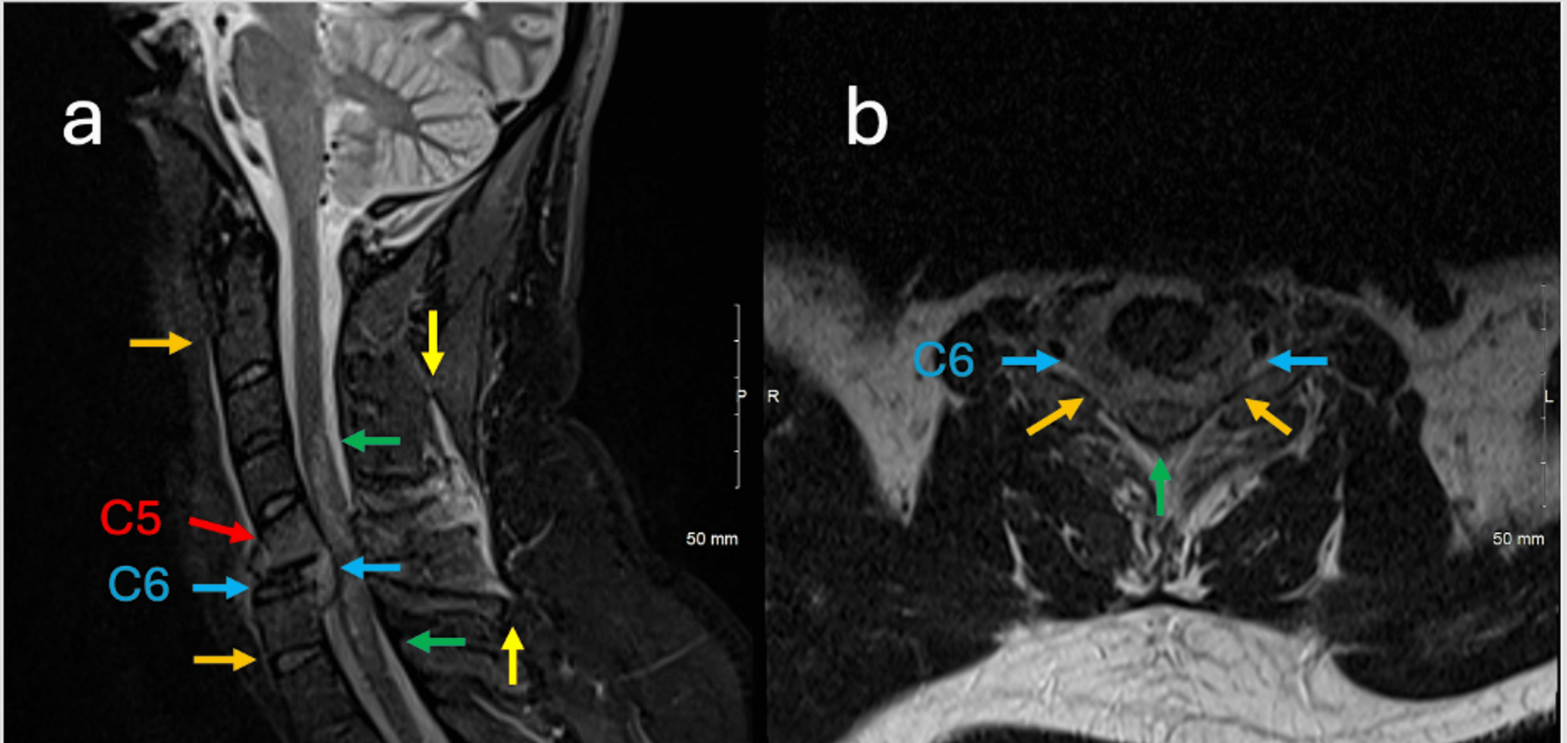
Sagittal STIR and Axial T2 MRI demonstrating metastatic paraganglioma with cervical vertebral collapse, ligamentous injury, and spinal cord compression. (a) Sagittal STIR image of the cervical spine demonstrating complete collapse of the C6 vertebral body (blue arrows) secondary to metastatic paraganglioma with destructive changes and retropulsion of the posterior vertebral body into the spinal canal. There is bone marrow edema from an acute anterior flexion teardrop fracture with mild retrolisthesis of C5 (red arrow). STIR bright cord signal abnormality (green arrows) from C4 down to T1 from cord impingement/compression is seen. There is also an interspinous and supraspinous ligamentous contusion secondary to the pathologic fracture (yellow arrows). Furthermore, there is a prevertebral hematoma (light orange arrows) from C2 to C7 from tearing of the anterior longitudinal ligament at the anterior inferior corner fracture of C5 (tip of red arrow). (b) Axial T2-weighted image at C5-C6 demonstrates circumferential collapse with retropulsion of tumour-infiltrated bone marrow into the spinal canal (green arrow) with cord impingement. There is also bilateral foraminal encroachment (light orange arrows).

MRI revealed collapse of the C6 vertebral body secondary to destructive tumor infiltration with superimposed extra-osseous extension of tumor/disc/hematoma complex into the prevertebral and anterior epidural spaces (Figure 1). There is significant compression at C5-C6 with resultant cord edema extended from upper C4 to upper T1 levels. Furthermore, an anterior flexion teardrop fracture of C5 with posterior ligamentous contusion was also identified.

**Figure 2:**
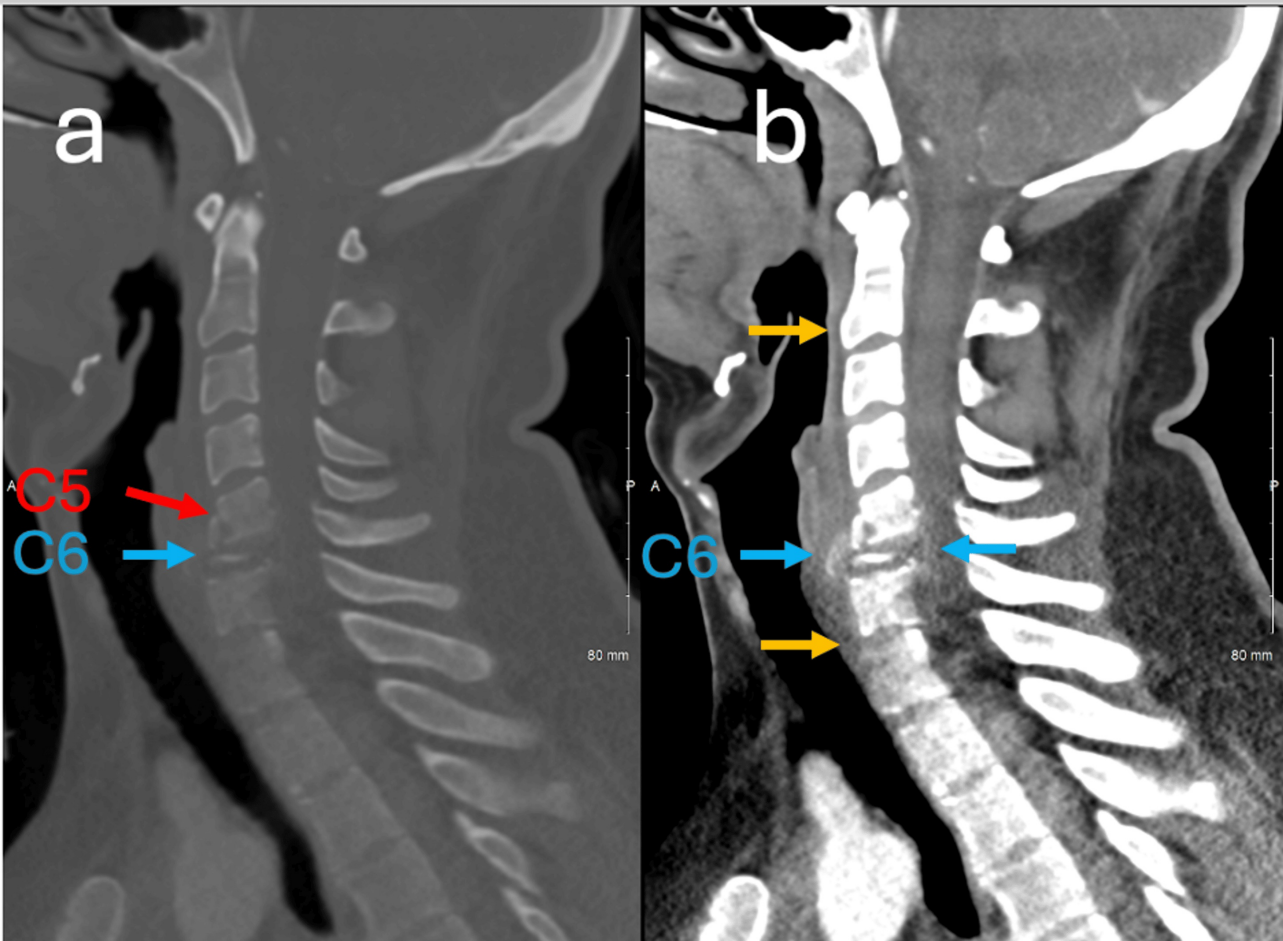
Sagittal bone and soft tissue window CT demonstrating C6 vertebral body destruction from metastatic paraganglioma with pathologic fracture and prevertebral hematoma. (a) Sagittal CT of the cervical spine with bone window demonstrates significant destructive changes of the C6 vertebral body secondary to proven metastatic paraganglioma (blue arrow) as well as flexion teardrop fracture with retrolisthesis of the C5 vertebral body with respect to the C6 upper endplate (red arrow). (b) Sagittal CT of the cervical spine with soft tissue window demonstrates C6 vertebral body collapse with circumferential expansion (blue arrows) resulting in canal narrowing. A small amount of prevertebral hematoma is also present (light orange arrows).

CT scan of the cervical spine showed an acute burst fracture of the C6 vertebra with nearly complete loss of height as well as a C5 anterior inferior corner fracture consistent with a known flexion teardrop fracture (Figure 2).

**Figure 3:**
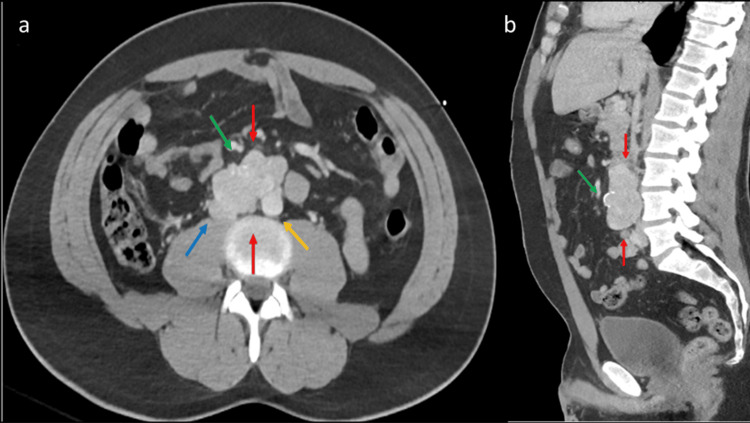
Axial and midsagittal CT of the abdomen and pelvis demonstrating retroperitoneal mass from metastatic paraganglioma. (a) Axial CT of the abdomen and pelvis demonstrates a well-circumscribed multilobulated soft tissue mass within the retroperitoneum (red arrows), intercalated between the aorta (light orange arrow) and inferior vena cava (IVC) (blue arrow). The lesion did not encase vessels and was therefore amenable to surgical resection. Note mild mass effect on the IVC and minimal calcification in the anterior tumor (green arrow). (b) Midsagittal CT of the abdomen and pelvis demonstrates the soft tissue density mass, presumed metastatic lymph node conglomerate from paraganglioma.

**Figure 4:**
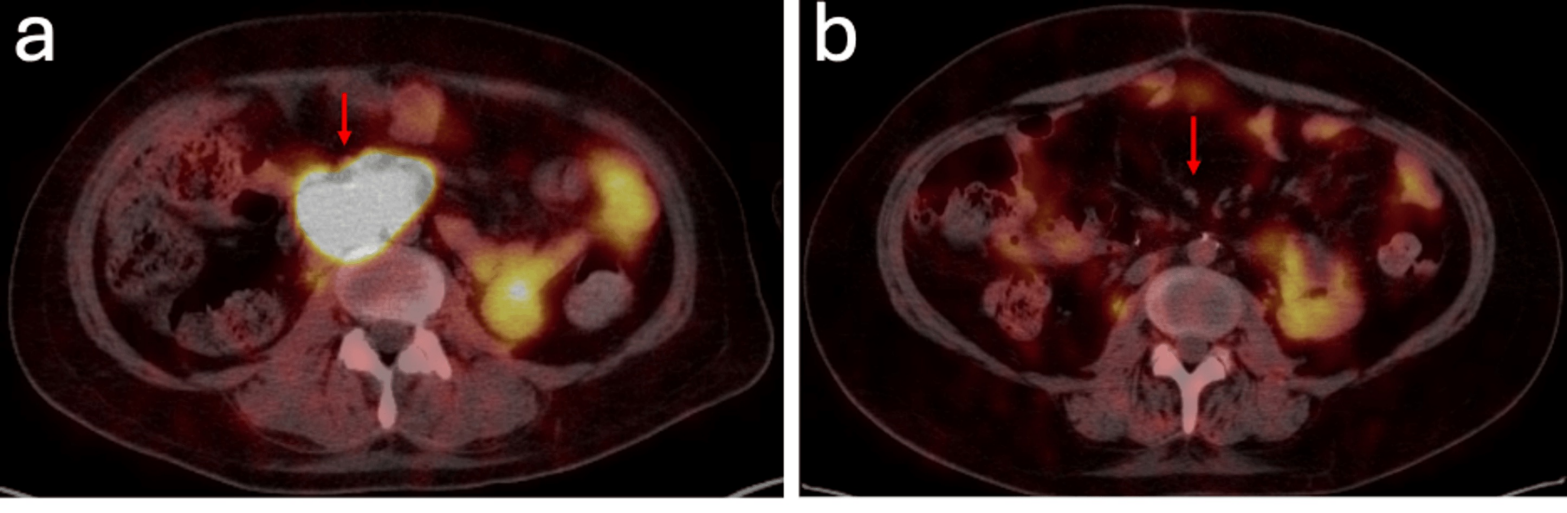
Pre and postoperative Cu-64 Dotatate PET/CT demonstrating resolution of radiotracer uptake following gross total resection of paraganglioma. (a) Axial images obtained from fused Cu-64 Dotatate PET/CT scan preoperatively demonstrate uptake of radiotracer in the tumor (red arrow). (b) Postoperative repeat imaging three months later demonstrated gross total resection of tumor without hypermetabolism to suggest residual or recurrent tumor, presumed paraganglioma. Normetanephrines were diminished postoperatively.

Further imaging obtained for trauma workup revealed an indeterminate lytic lesion in the left scapula and right iliac bone, and an irregular, partially calcified aortocaval mass, likely a nodal conglomerate, measuring 3.5 cm anteroposterior (AP) × 4.4 cm transverse (TR) × 7.2 cm craniocaudal (CC) (Figures 3, 4). Upon further questioning, the patient’s father revealed that the patient underwent abdominal surgery for mass resection at a young age. However, the patient had not received further treatment or follow-up, and the pathology was unknown to the family.

Surgical intervention

The patient was taken emergently to the operating room for decompression and stabilization of his C6 pathologic fracture with active spinal cord compression secondary to presumed hematoma. C6 corpectomy was performed in a standard fashion. Intra-operatively, a large purple soft tissue mass was identified and biopsied. Frozen pathology was consistent with unspecified lymphoma. The patient was extubated post-operatively and transferred to the intensive care unit for further management of his acute spinal cord injury.

Histopathological examination

**Figure 5:**
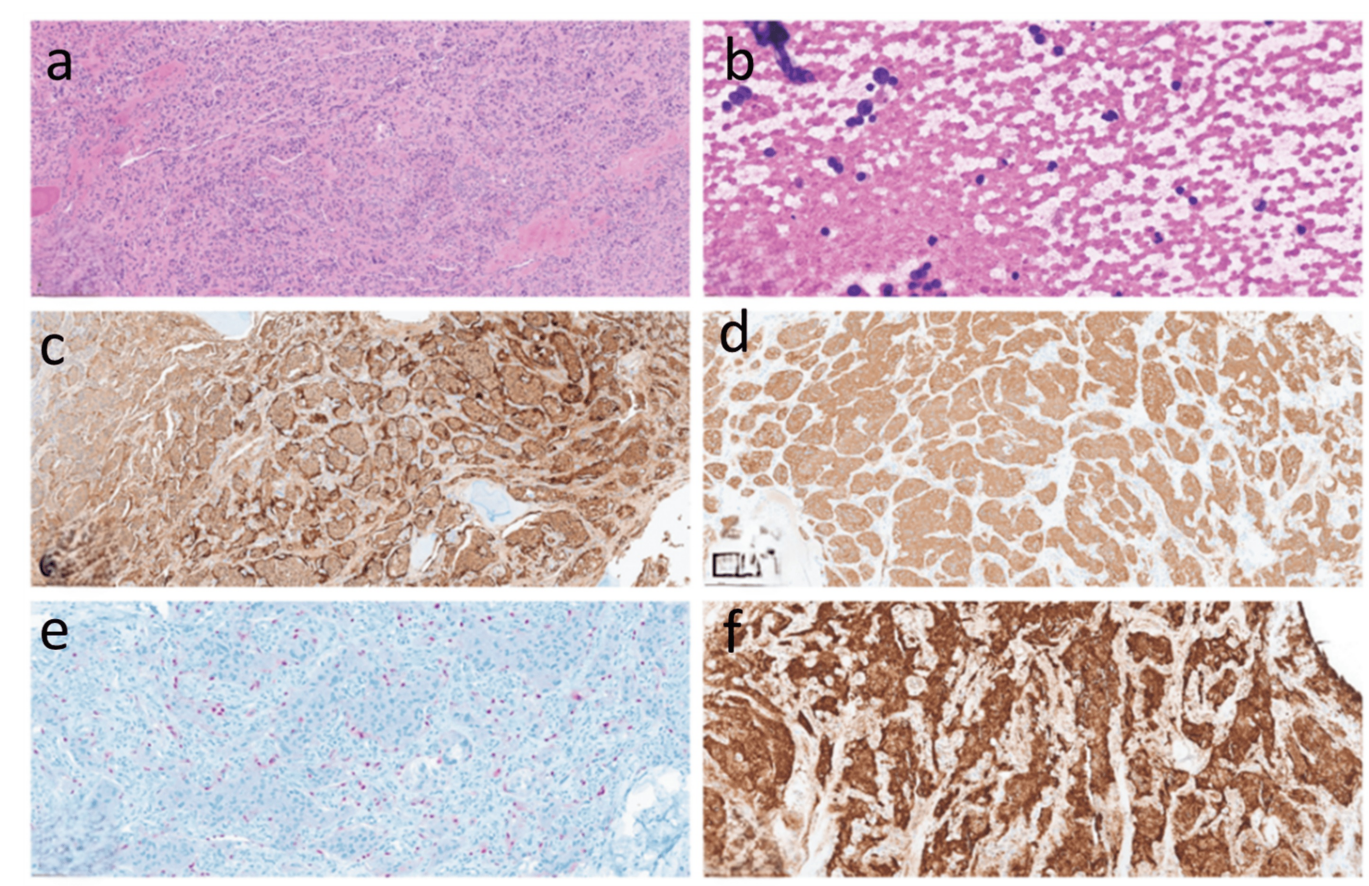
Histopathologic and immunohistochemical features of paraganglioma. (a) Hematoxylin and eosin (H&E) sections show classic paraganglioma morphology with monomorphic cells and eosinophilic cytoplasm arranged in a nested (Zellballen) pattern separated by fibrovascular septae containing sustentacular cells. (b) H&E smear highlights the characteristic salt-and-pepper “neuroendocrine type” chromatin. (c) Immunohistochemistry demonstrates diffuse positivity for S100. (d) Immunohistochemistry demonstrates diffuse positivity for synaptophysin. (e) Epithelial membrane antigen and neurofilament were negative. Sustentacular cells stained positive for SOX10 and CD34, highlighting the blood vessel. (f) SDHB expression was retained.

Hematoxylin and eosin-stained (H&E) sections of the tumor samples revealed the typical paraganglioma morphology, monomorphic cells with eosinophilic cytoplasm arranged in a nested (Zellballen) pattern separated by fibrovascular septae containing sustentacular cells (Figure 5a). H&E-stained smear highlighted the nuclear salt and pepper neuroendocrine type chromatin (Figure 5b). Immunohistochemistry studies further supported the diagnosis with diffuse positivity for S100 (Figure 5c) and synaptophysin (Figure 5d). Epithelial membrane antigen (EMA) and neurofilament were negative. The sustentacular cells were SOX10 positive (Figure 5e), while CD34 highlighted the blood vessel. SDHB was retained in the tumor cells (Figure 5f), and molecular studies further confirmed the absence of mutation. Malignant PG has no established histopathological criteria, except in the presence of documented metastasis.

Postoperative course and treatment

The patient’s blood pressure was augmented with norepinephrine to achieve mean arterial pressures greater than 85, per standard of care for traumatic spinal cord injury. Leg wraps and abdominal binders were placed when the patient was sitting up to aid in venous return. He also underwent standard bowel and bladder care, given the level of his injury. On post-operative day (POD) two, the patient’s respiratory status declined, requiring intubation. Following intubation, the patient remained neurologically stable. On POD 5, the patient started experiencing cyclic fevers, diaphoresis, tachycardia and extreme hypertension with systolic blood pressure (SBP) in the 200s. The patient was initiated on labetalol with a target systolic blood pressure below 160 mmHg, though this achieved a limited effect. On POD 6, pathology confirmed metastatic paraganglioma, prompting consultation with oncology and endocrinology due to concern for a secreting tumor. Biochemical evaluation demonstrated markedly elevated metanephrine levels of 44 nmol/L and normetanephrine levels of 3,411 ng/L, findings consistent with a predominantly noradrenaline-secreting paraganglioma. Labetalol was immediately terminated, and doxazosin was started. However, the patient continued to have extreme hypertension requiring the addition of other anti-hypertensive agents, including amlodipine, propranolol and clonidine. Once medically stabilized, the patient underwent tracheostomy and percutaneous gastrostomy tube placement prior to discharge to rehabilitation for his acute spinal cord injury.

## Discussion

PGs are rare endocrine tumors originating from neural crest-derived chromaffin cells [1]. They can typically arise in one of three common locations: (i) the adrenal medulla, where they are called pheochromocytomas or intra-adrenal sympathetic paragangliomas, (ii) the extra-adrenal sympathetic paraganglia located below the diaphragm, and (iii) parasympathetic anterior thoracic and head and neck paraganglia [11,12]. Only 10% of PGs arise extra-adrenally [4]; however, both intra-adrenal and extra-adrenal sympathetic PGs can secrete catecholamines [13] inducing hypertension, headaches, and paroxysms [14]. Standard management of malignant PGs includes alpha-adrenergic blockade followed by beta-blockade to prevent systemic hypertension, volume replacement as patients are often vasoconstricted and hypovolemic, and surgical resection [15]. Once the secreting mass is removed, patients may experience orthostatic hypotension and arrhythmias, which can be medically managed. Given the potential hemodynamic instability of these patients, careful perioperative optimization and management must be performed with collaboration from oncologists and endocrinologists. Germline mutations in the succinate dehydrogenase complex subunit B (SDHB) gene are associated with poor outcomes and shorter survival in patients with malignant PG [16].

PGs exhibit minimal pleomorphism, mitotic activity [8] and low malignant potential, with only 10% spreading to distant sites [3,17] such as the bone, liver, lungs, and lymph nodes [1]. The occurrence of vertebral metastasis is extremely uncommon [17] and is typically the result of local spread. Even rarer is the involvement of the cervical spine [3,4]. 

Acute cervical spinal cord injuries often result in neurogenic shock, leading to hypotension, bradycardia, and autonomic dysreflexia secondary to disruption of sympathetic signaling. The aberrant systemic regulation can last from 1-6 weeks [18]. Standard of care for spinal cord injury addresses dysregulation through blood pressure and heart rate augmentation with vasopressors and intermittent compressive devices to assist in venous return. Norepinephrine is the first-line therapy for neurogenic shock management in cervical spine injuries, given its alpha and beta-adrenergic receptor activation [19].

Upon arrival, our patient exhibited signs of neurogenic shock, including hypotension and bradycardia with a heart rate that was sustained in the 50s. Therefore, any previous systemic effects and symptoms of his malignant PG were masked. However, when his neurogenic shock resolved one week after initial presentation, the patient developed symptoms of catecholamine excess, including malignant hypertension, cyclical fevers, diaphoresis and tachycardia. Once a tissue diagnosis was confirmed, the patient was started on appropriate systemic therapy for management of catecholamine excess. Following stabilization, the patient was able to be discharged to rehabilitation. Literature review revealed 14 additional cases of cervical vertebral metastasis and pathologic fractures. The average age of diagnosis was 43, with males affected more predominantly than females (10M:5F), and the average time of metastasis was 8.6 years (Table 1). The data highlights the diagnostic uniqueness of our current patient, who presented at the age of 25, with a 19-year interval between metastases. Of these 14 cases, eight patients had signs and symptoms consistent with cervical myelopathy. Based on this limited data, patients with metastasis to the cervical spine are at high risk for spinal cord compression and worsening injury in the setting of mild trauma, as experienced in our case. When applicable, early collaboration with appropriate specialists is recommended to guide surgical management, supportive care to avoid an acute hypertensive crisis.

**Table 1:** 15 cases of cervical body metastases from paragangliomas including the present case. Summary of published cases of cervical spine metastasis from paragangliomas, including the present case. Note: M: male; F: female; yr: year(s); mets: metastasis.

Reference	Age (yr)	Sex	Primary tumor	Time to develop mets (yr)	Symptoms
Lehmen et al. 2010 [3]	71	M	Unknown (presented with bilateral carotid body: right carotid body resected, left intact)	9	Progressive neck and thoracic back pain, left upper and lower extremity weakness
Kapetanakis et al. 2018 [4]	52	M	Jugular foramen	3	Increasing neck pain and upper extremity weakness
Osborn et al. 1986 [5]	47	F	Right glomus jugulare	4	Pain after a minor injury to the neck
Sasaki et al. 2013 [6]	72	M	Neck	5	Bilateral shoulder pain, severe neck pain, and weakness of the upper extremities
Lau et al. 2013 [8]	47	M	Abdomen	0.5	Progressive midline neck pain, expired 52 months after diagnosis of his metastatic disease
Brodkey et al. 1995 [10]	39	M	Left carotid body	7	Back, neck, and arm pain; normal exam
Brodkey et al. 1995 [10]	56	M	Left neck	15	Pain and numbness right hand
Brodkey et al. 1995 [10]	19	F	Left carotid body	2	C5 sensory level, severe quadriparesis
Brodkey et al. 1995 [10]	38	F	Right jugulotympanic	23	Right leg weakness, Babinski’s sign, and clonus
Brodkey et al. 1995 [10]	54	M	Retroperitoneal supra-adrenal	14	Mild central cord syndrome
Lázaro et al. 2003 [14]	29	F	Left carotid body	5	Cervico-brachial pain
North et al. 1990 [17]	28	F	Left carotid body	9	Doing well thirteen months after treatment
Coulson et al. 1970 [20]	43	M	Right neck	14	Low back and leg pain
Yamaguchi et al. 2003 [21]	27	M	Cardiac pheochromocytoma	N/A	Severe neck pain, died of metastasis to the brain
Present case	25	M	Abdomen	19	Abdominal pain at the age of six years and underwent a surgery; pathologic cervical spine fracture and spinal cord injury after trauma at the age of 25 years

## Conclusions

Our experience demonstrates the rare clinical anomaly of metastatic paraganglioma to the cervical spine, resulting in pathological fracture and acute spinal cord injury. This case highlights the diagnostic complexity and therapeutic challenges of metastatic paraganglioma. Furthermore, this report adds to the sparse literature on cervical spine dissemination and may help guide evaluation and clinical decision-making practices in similar cases.
